# Green Surfactant-Free Synthesis of Mesoporous Silica Materials via a Biomass-Derived Carboxylic Acid-Assisted Approach

**DOI:** 10.3390/nano16010045

**Published:** 2025-12-29

**Authors:** Ivalina Trendafilova, Stela Grozdanova, Ágnes Szegedi, Pavletta Shestakova, Yavor Mitrev, Bogdan Ranguelov, Daniela Karashanova, Margarita Popova

**Affiliations:** 1 Institute of Organic Chemistry with Centre of Phytochemistry, Bulgarian Academy of Sciences, 1113 Sofia, Bulgaria; ivalina.trendafilova@orgchm.bas.bg (I.T.); stela.grozdanova@orgchm.bas.bg (S.G.); pavletta.shestakova@orgchm.bas.bg (P.S.); yavor.mitrev@orgchm.bas.bg (Y.M.); 2 National Centre of Excellence Mechatronics and Clean Technologies, 8 bul. Kliment Ohridski, 1756 Sofia, Bulgaria; 3 HUN-REN Research Centre for Natural Sciences, Institute of Materials and Environmental Chemistry, Magyar tudósok krt. 2., 1117 Budapest, Hungary; szegedi.agnes@ttk.hu; 4 Institute of Physical Chemistry, Bulgarian Academy of Sciences, 1113 Sofia, Bulgaria; rangelov@ipc.bas.bg; 5 Institute of Optical Materials and Technologies, Bulgarian Academy of Sciences, 1113 Sofia, Bulgaria; dkarashanova@yahoo.com

**Keywords:** sitheerogelsofcarboxylic acids, pore size control, porogen recycling

## Abstract

This study presents a novel approach for synthesizing porous silica materials using various biomass-derived carboxylic acids as non-surfactant, eco-friendly porogens. Different carboxylic acids were selected, and their influence on the properties of the final materials was systematically investigated. The silica synthesis was performed using only the intrinsic acidity of carboxylic acids without pre-hydrolysis of the silica precursor - tetraethyl orthosilicate (TEOS). Citric and tartaric acids had a favorable effect on the formation of mesoporous silica, whereas the oxalic, ascorbic, maleic, and mandelic acids led to the formation of microporous silica. The optimal synthesis compositions and parameters were thoroughly investigated. A mesoporous silica with a uniform pore size was prepared using tartaric acid, and the pore size was controlled by the drying temperature. Template removal via water extraction yielded silica materials with superior textural properties compared to conventional high-temperature calcination, while allowing the recovery and reuse of organic acids. Our results confirm that using carboxylic acids can be efficiently and economically applied for the controlled synthesis of mesoporous silica suitable for different applications, such as adsorption, drug delivery, and catalysis.

## 1. Introduction

Porous silica xerogels have emerged as versatile materials with significant potential across a broad range of applications, including catalysis, adsorption, separation, thermal insulation, optics, and controlled drug release [1,2,3]. These materials are characterized by their low density, high surface area, and tunable pore architecture, which can range from microporous (<2 nm) to mesoporous (2–50 nm) or even macroporous (>50 nm) regimes, depending on the synthesis conditions and templating agents employed [4]. Among the various classes of porous materials, mesoporous silica xerogels offer an optimal combination of accessibility, diffusivity, and structural stability, making them particularly valuable in product separation, molecule adsorption, gas storage, heterogeneous catalysis, and pollutant removal [5].

One of the most developed methods for synthesizing silica gels is the sol-gel process. This process allows control over the structure and morphology of the materials [6]. In the sol-gel process, silicon alkoxides (e.g., tetraethyl orthosilicate, TEOS) undergo hydrolysis and condensation reactions under acidic or basic conditions to form a three-dimensional silica network [7]. To tailor the porosity of the obtained materials, surfactants or polymeric templates, such as cetyltrimethylammonium bromide (CTAB), Pluronic P123, and polyethylene glycol (PEG), are commonly introduced into the precursor solution [8]. Depending on the template used, its concentration, and the pH, materials with different pore sizes and shapes, as well as pore system structures, can be obtained [9,10, 11]. However, these templates are associated with significant drawbacks, including high cost, poor biodegradability, and the requirement of harsh conditions (e.g., high-temperature calcination or solvent extraction) for their removal [12]. These factors not only increase the environmental footprint of the process but also limit the scalability and sustainability of silica xerogel production.

In response to these challenges, there has been growing interest in developing green synthesis strategies that utilize renewable and eco-friendly templating agents. Numerous studies have demonstrated that various classes of renewable or biologically derived compounds can serve as effective templating agents for sustainable silica synthesis. These include surfactants and non-surfactants obtained from renewable resources (e.g., fatty acids, amino acids, and sugars), cleavable surfactants such as betaine and glycine betaine, biosurfactants produced by microorganisms or isolated enzymes, phospholipids, sophorolipids, and polymers and colloidal systems derived from natural sources such as chitosan, chitin colloids, and cellulose-based nematic liquid crystals [13,14]. Among this huge variety of potential porogens, biomass-derived organic acids stand out as environmentally friendly and cost-effective owing to their natural abundance, biodegradability, and functional diversity [15,16,17]. These organic acids contain multiple carboxyl and hydroxyl groups capable of forming hydrogen bonds or ionic interactions with hydrolyzed silica species, thereby influencing the condensation rate, particle growth, and pore formation process [18]. Previous studies have shown that organic acids can play multiple roles in silica synthesis, including pH modulation, complexation with silanol groups, and supramolecular self-assembly, all of which contribute to the development of hierarchical pore networks [19,20]. For instance, citric acid has been demonstrated to act as a chelating agent and steric barrier during sol–gel synthesis, leading to increased mesoporosity and homogeneity in xerogels [21]. Mesoporous silica materials prepared in the presence of carboxylic acids as templates have shown interconnected worm-like pores. Despite not having a long-range order like other mesoporous silicas, such as MCM-41 and SBA-15, the branched structure of these materials could improve the access to active sites in the silica framework [22]. Oxalic acid in combination with glycerol can be used as a drying control chemical additive in the synthesis of iron-doped silica xerogels, helping to improve the structural properties of the xerogel [23]. Another study reported the use of tartaric, malic, and citric acids as pore-forming agents added to prehybridized with mineral acid silica precursors [24]. Furthermore, their thermal decomposition behavior enables facile template removal under relatively mild conditions compared to conventional calcination techniques. Another method for template removal is extraction. Because organic acids are water-soluble, the templates can be easily removed by a simple washing procedure. The water extraction procedure also allows the regeneration and reuse of the acids.

Despite these promising attributes, systematic investigations of biomass-derived organic acids as structure-directing agents in silica xerogel synthesis remain limited. Most previous research has focused on aerogels, whose preparation requires a supercritical drying step and involves high processing costs. In contrast, xerogels offer a more scalable and energy-efficient alternative because of their simpler ambient drying process; however, their porosity is often compromised without proper templating control [25]. Moreover, there is a lack of comparative studies assessing the impact of different organic acids on the final textural properties of xerogels, especially regarding their influence on surface area, pore size distribution, and structural integrity after template removal.

In this study, a novel approach for the synthesis of porous silica xerogels was developed, utilizing carboxylic acids as both drying control and/or porogenic agents. The xerogels were prepared without the prehydrolysis step of the silica precursor, commonly used in other published studies, relying on the intrinsic acidity of the organic acids to adjust the pH, and using water as asolvent in most cases. For the first time, different carboxylic acids were used in the preparation of porous silica, and their influence on the textural properties of the final product was studied. The synthesis conditions and template removal methods were systematically optimized to obtain porous silica materials with superior structures, and their impact was evaluated. Furthermore, the feasibility of recovering and cycling porogenic agents was evaluated. The overall process was further optimized considering energy and cost efficiency, reduced processing time, and minimized environmental impact.

## 2. Experimental

### 2.1. Materials

**Scheme 1:**
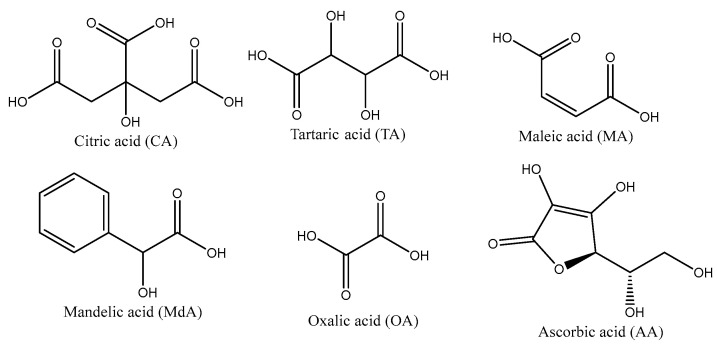
Structure of carboxylic acids used as porogens in silica synthesis.

Tetraethyl orthosilicate (TEOS, Sigma Aldrich, St. Louis, MO, USA) was used as the silica source. The carboxylic acids used as porogens were citric acid (CA, local vendor, Sofia, Bulgaria), oxalic acid (OA, local vendor), ascorbic acid (AA, Thermo Scientific Chemicals, Waltham, MA, USA), tartaric acid (TA, Thermo Scientific Chemicals), maleic acid (MA, Thermo Scientific Chemicals), and mandelic acid (MdA, Thermo Scientific Chemicals). The chemical structures of the acids used are shown in Scheme 1.

### 2.2. Synthesis of Mesoporous Silica

In a typical synthesis, the carboxylic acid (0.02 or 0.06 M) was dissolved in 50 mL of distilled water, and 4.17 g (0.02 M) TEOS was added dropwise to the solution (water/TEOS molar ratio = 138.5). Various acid-to-TEOS molar ratios (1 and 3) were used to determine the optimal synthesis composition. The mixture was stirred at room temperature for 120 min and then dried at 30, 60, or 90 °C under atmospheric pressure. Silica xerogels were labeled as MS-Acid-X-x, where MS means mesoporous silica, X indicates the drying temperature (30, 60, or 90 °C), and x represents the acid: TEOS molar ratio (1 or 3). All syntheses were conducted in water, except for mandelic acid, which has low water solubility. In this case, ethanol was used as the solvent. Two methods were employed to remove the organics: high-temperature calcination and water extraction. Calcination was performed at 550 °C for 5 h at a heating rate of 3 °C/min in stagnant air. Complete removal of the acids was accomplished through two successive extraction cycles, each involving vigorous stirring of a suspension containing 1 g of silica–acid composite in 100 mL distilled water for 1 h. The resulting template-free materials were designated as MS-Acid–X-x-C (calcined) and MS-Acid–X-x-W (water-extracted). For a more detailed investigation of the synthesis mechanism, the non-calcined, as-prepared silica-organic acid composites were labeled with P″. In addition, some samples were synthesized using recycled (water-extracted) carboxylic acid to assess the reusability of the acid in the second cycle of mesoporous silica xerogel preparation. This material was designated MS-Acid-X-x-C–R. Two additional samples were prepared for comparison with the above-described materials: one with TEOS in water without adding any acid, and another prepared using an HNO_3_ solution with a pH = 1.2. These samples were dried at 60 °C and denoted as MS-TEOS-60 and MS- HNO_3_-60, respectively.

### 2.3. Characterization Techniques

X-ray diffraction patterns were recorded using a Philips X’Pert MD diffractometer (Malvern Panalytical, Almelo, The Netherlands) with monochromatized CuKα radiation (40 kV, 35 mA). Patterns were collected from 3 to 50°2θ, by 0.04° step size for 4 s each. The ICDD PDF2 database was used to identify the crystalline phases.

The textural properties of the materials, such as the specific surface area, pore volume, and pore size distribution, were determined from N_2_ physisorption isotherms collected at −196 °C using AUTOSORB iQ-C-MP-AG-AG (Quantachrome Instruments, Anton Paar, Boynton Beach, FL, USA). The samples were pretreated at 150 °C under vacuum for 10 h. The specific surface area was calculated from the adsorption isotherm branch at a relative pressure of 0.05 to 0.21 using the Brunauer-Emmett-Teller (BET) equation [26]. The total pore volume was estimated based on the amount of absorbed gas at p/p_0_ = 0.98, according to the Gurvich rule [27]. Barrett-Joyner-Halenda (BJH) method was applied for evaluation of the pore size distribution, and the maximum pore size was calculated from the desorption branch [28]. The micropore volume and mesopore surface area were calculated using the t-plot method.

NMR was used to determine the structural characteristics of the obtained materials, and the spectra were recorded on a Bruker Avance III HD 600 NMR spectrometer (Bruker, Karlsruhe, Germany) operating at 599.98 MHz ^1^H frequency (119.21 MHz for ^29^Si), using a 4 mm solid-state i-CP/MAS dual ^1^H/^31^P-^15^N probehead. The samples were packed in 4 mm rotors (Zr_2_O) and spun at a magic angle spinning (MAS) rate of 10 kHz. The quantitative direct-excitation ^29^Si NMR spectra were acquired with a single-pulse sequence, 90° pulse length of 2.3 s, time domain data points of 3 K, spectrum width of 71 kHz, and relaxation delay of 60 s. The spectra were zero-filled to 16 K data points and processed with an exponential window function (line-broadening factor 20) before Fourier transformation. The ^1^H-^29^Si cross-polarization MAS (CP MAS) spectra were acquired using the following experimental parameters: ^1^H excitation pulse of 2.5 µs, contact time of 5 ms for ^29^Si, and a relaxation delay of 5 s. The ^1^H SPINAL-64 decoupling scheme was used during the acquisition of all CP experiments. The number of scans was optimized for each sample, but typically 1024 scans were accumulated for the direct excitation ^29^Si experiments and 1024–4096 scans were accumulated for the ^1^H-^29^Si CPMAS experiments. The chemical shift referencing of the ^29^Si spectra was performed using octakis (trimethylsiloxy)silsesquioxane powder (Q8M8, δ ^29^Si = 11.77 ppm).

The organic content and efficiency of the template removal procedures were monitored by temperature-programmed oxidation–thermogravimetric analysis (TPO-TGA) performed on an STA449F5 Jupiter instrument (NETZSCH Gerätebau GmbH, Netzsch, Germany). The studied sample was placed in a ceramic crucible and heated in air (50 cm^3^/min) at a rate of 10 °C/min up to 600 °C with a final hold-up of 1 h.

The morphology and qualitative elemental analysis of the samples were obtained using a state-of-the-art modern field-emission scanning electron microscope (JEOL IT800SHL, JEOL Ltd., Tokyo, Japan) using both secondary and backscattered electron detectors placed within the in-chamber and in-lens microscope columns. The samples (as powders) were dispersed onto conductive carbon tape, and no evaporation of Au or C was used.

The morphology and microstructure of the studied materials were observed using a JEOL JEM 2100 transmission electron microscope (JEOL Ltd., Tokyo, Japan) in bright-field TEM (BF-TEM) mode at an accelerating voltage of 200 kV. Preliminary preparation, which consisted of suspending silica powder in ethanol and sonication for 3 min, was applied. Micro-quantities of the sample were then dropped onto standard copper TEM grids covered with amorphous carbon. The samples were dried under ambient conditions without any further treatment.

The carboxylic acid-silica interactions were investigated using attenuated total reflectance (ATR) infrared (IR) spectroscopy. The ATR-IR spectra were acquired using a Bruker Invenio R spectrometer equipped with a diamond crystal PIKE Technology (Fitchburg, WI, USA) ATR accessory. The samples were studied in the solid state by directly depositing the powder on the diamond surface. The spectra were recorded in the frequency range of 4000–600 cm^−1^ with 200 scans at a resolution of 2 cm^−1^, with air as the background. The spectra were baseline-corrected, and the atmospheric moisture was removed using a standard correction algorithm.

## 3. Results and Discussion

Porous silica materials were characterized using physicochemical methods at each stage of the preparation process. The effects of the porogen type and amount, synthesis conditions, and porogen removal methods were systematically investigated. The formation and properties of the resulting organic–inorganic composites were analyzed using powder X-ray diffraction (XRD) and solid-state nuclear magnetic resonance (NMR). Textural characteristics such as the specific surface area, pore volume, and pore type were evaluated using nitrogen physisorption. The morphology and surface features of the particles were examined using scanning electron microscopy (SEM) and transmission electron microscopy (TEM). The organic phase content of the composite materials, both before and after porogen removal, was monitored using temperature-programmed oxidation (TPO), allowing the assessment of initial and residual organics following calcination or extraction.

### 3.1. Influence of the Synthesis Conditions and Composition

#### 3.1.1. Effect of Drying Temperature

First, the influence of drying temperature on xerogel synthesis was evaluated using materials prepared in the presence of three representative carboxylic acids from the six presented in Scheme 1, namely citric, oxalic, and tartaric acids. The drying temperature is a critical factor in the synthesis of silica xerogels, as it can influence both the crystallization behavior of the organic acid templates and the kinetics of silica precursor condensation. Drying was carried out at three different temperatures: 30, 60, and 90 °C using an acid-to-TEOS molar ratio of 3.

**Figure 1:**
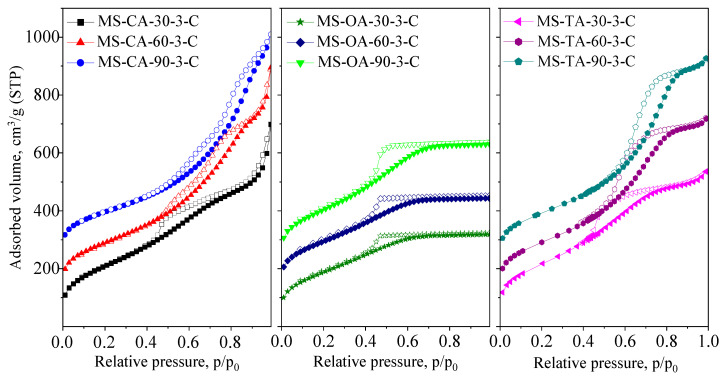
N_2_ physisorption isotherms of silica xerogels prepared using citric (CA), oxalic (OA), and tartaric acids (TA) at various drying temperatures (Acid: TEOS = 3). The isotherms of the samples dried at 60 °C and 90 °C were shifted by 100 and 200 cm^3^/g, respectively. The adsorption and desorption branches are represented by filled and empty symbols, respectively.

The pore systems of the xerogels were studied using N_2_ physisorption. The isotherms are shown in Figure 1, and the textural parameters are summarized in Table 1. The isotherms are of type IV, which is typical of mesoporous silica materials, and they exhibit H2/H3 hysteresis loops. No micropore volume was determined using the t-plot method.

A general trend was observed: as the drying temperature increased, the pore volume also increased. In most cases, this corresponded to an increase in the pore size and a narrower pore size distribution. This tendency was most pronounced in the samples synthesized with tartaric acid, as evidenced by the shift of the isotherms to higher relative pressures and the increased parallelism of the hysteresis loop branches. A similar phenomenon was observed in samples prepared with citric acid; however, in this case, a bimodal pore structure appeared at higher drying temperatures (3.9/6.7 nm for the material prepared at 60 °C and 4.6/8.6 nm for the material prepared at 90 °C).

The isotherms exhibited an H3-type hysteresis loop (without a plateau), indicating the presence of larger pores owing to the formation of interparticle porosity. The textural mesoporosity of the MS-CA samples (Figure 1) is also evident, as indicated by a steep increase in the adsorbed volume at high relative pressures, reflecting the formation of mesopores within the voids between agglomerated silica particles. In contrast, the xerogels synthesized with oxalic acid showed minimal dependence on the synthesis temperature. The shape of the isotherms remained consistent, reflecting a broad pore size distribution, with H2 type hysteresis loop. Only the sample dried at 90 °C exhibited a significant increase in pore volume.

The increased pore volume can be attributed to the accelerated solvent evaporation at elevated temperatures, which may promote the rapid formation of larger organic acid crystals. At the same time, a higher evaporation rate decreases the solvent viscosity, resulting in a lower capillary pressure gradient. Consequently, gel shrinkage was hindered during the drying stage, leading to an increase in the pore diameter. Larger pore diameters and lower interfacial tensions reduce the capillary pressure on the pore walls at the critical point, helping to preserve the mesopores.

These findings highlight the critical role of thermal conditions in modulating the porosity and textural properties of the synthesized material.

The crystalline phases of the silica-carboxylic acid composites after drying at different temperatures were studied using X-ray powder diffraction. The crystalline forms detected on the external surface and the related ICDD card numbers used for their identification are summarized in Table S1 (Supporting Information). In the low-angle XRD patterns of the silica-organic acid composites, characteristic reflections of ordered pore structures were not observed. In the high-angle patterns (Supporting Information, Figure S1A), crystalline forms of carboxylic acids can be observed in addition to the amorphous halo of silica at approximately 25° 2θ. Compared to the initial carboxylic acids dried at different temperatures, the composites showed the original forms of the acids in most cases, with some exceptions. For example, oxalic acid preparations exhibit the hydrate forms of the acid at lower temperatures and a mixture of hydrate and anhydrous oxalic acid at higher temperatures. Tartaric acid composites show mixtures of L^+^ and D^−^ enantiomers at higher temperatures with a higher ratio of L^+^. Comparing the same MS-acids dried at different temperatures, some changes were observed in accordance with the nitrogen physisorption data. The intensity of the crystalline acid phase decreased proportionally with the increase in the mesoporous character of the silica samples. The embedding and amorphization of the acid phase were enhanced by increasing the mesopore volume; therefore, the amount of separate crystalline phases decreased.

#### 3.1.2. Effect of Acid-to-TEOS Molar Ratio

To optimize the synthesis protocol, the molar ratio of tetraethyl orthosilicate (TEOS) to carboxylic acid was varied systematically. The samples were prepared using acid-to-TEOS molar ratios of 1 and 3. Fine-tuning the acid concentration is critical, as the formation of a well-defined nanocomposite requires the silica phase to be highly dispersed—ideally at the nanometer scale—to facilitate the development of a rigid and interconnected network.

As summarized in Table 2, a consistent trend was observed across all samples, regardless of the organic acid used.

**Figure 2:**
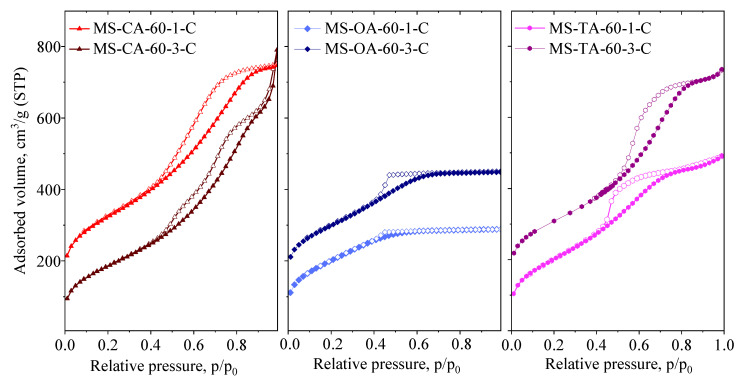
N_2_ physisorption isotherms of silica xerogels prepared with different acid-to-TEOS molar ratios (dried at 60 °C). The Acid:TEOS = 3 samples were shifted by 100 cm^3^/g values.

Similarly, the effect of drying temperature, increasing the acid content resulted in a decrease in the specific surface area, accompanied by an increase in the total pore volume, indicating the formation of larger pores [29]. By analyzing the isotherms (Figure 2), it can be observed that at lower concentrations of carboxylic acids, the isotherm shapes remain unchanged (all exhibit an H2-type hysteresis loop); however, the pore size distribution shifts toward smaller values. A similar phenomenon was reported by Pang et al. [30] for maleic and citric acids, where both the pore size and volume increased with higher template concentrations. In the case of the oxalic acid (OA) samples, the pore system consisted of mesopores with narrower pore entrances than those formed with other carboxylic acids. At lower oxalic acid contents, the pore size shifted into the supermicropore range (0.7–2 nm), whereas larger mesopores appeared only at higher oxalic acid concentrations. At lower acid contents, the bimodal pore system observed in citric acid (CA) samples becomes less pronounced owing to a shift in the dominant pore size from 6.7 to 5.4 nm. In terms of macroscopic morphology, the particle shape of MS-CA-60-3-C appears to differ notably, as indicated by the emergence of textural mesoporosity. The positive effect of the tripled carboxylic acid content was most evident in the TA sample, where a significant increase in both pore volume and uniformity was observed.

The results described above demonstrate a clear and consistent trend in the influence of the acid-to-TEOS molar ratio, highlighting its potential for the precise and effective control of the structural and textural properties of the resulting silica materials. This tunability offers significant advantages in tailoring material characteristics to meet specific application requirements.

Further increasing the acid: TEOS ratio to 1:6 led to a significant decrease in the surface area of the obtained material by 36% in comparison to the sample prepared with an acid: TEOS ratio of 1:1 (Table S2). Therefore, for all further experiments, samples prepared with an acid:TEOS ratio of 1:1 were selected.

#### 3.1.3. Type of Acid

A variety of carboxylic acids were investigated as potential cost-efficient and eco-friendly porogens or drying control agents for the synthesis of porous silica xerogels. To evaluate the influence of the acid type on the textural and physicochemical properties, the synthesis conditions were kept consistent across all samples: a drying temperature of 60 °C and an acid-to-TEOS molar ratio of 1. All samples were prepared in water, except for the sample prepared with mandelic acid, which was dissolved in ethanol.

**Figure 3:**
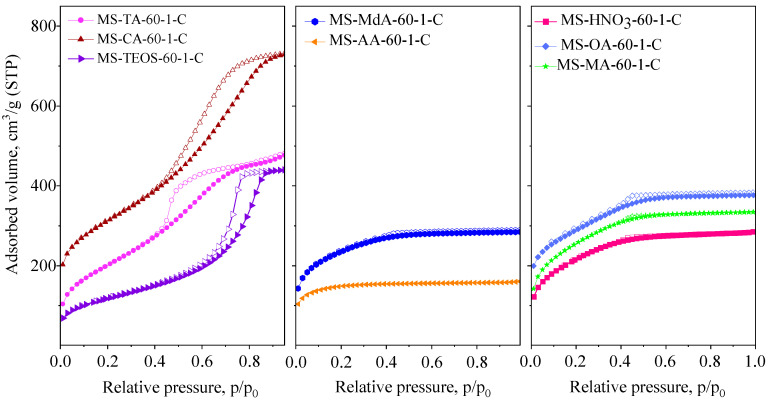
N_2_-physisorption isotherms of silica xerogels prepared with different types of carboxylic acids (acid: TEOS = 1, dried at 60 °C, and calcined at 550 °C). The isotherms of the MS-CA-60-1-C and MS-OA-60-1-C samples were shifted by 100 cm^3^/g.

The specific surface area, total and micropore volumes, and mesopore surface area of the xerogels prepared using different acids were calculated from the nitrogen physisorption isotherms (Figure 3), and the corresponding data are summarized in Table 3.

The specific surface area and pore volume were significantly influenced by the type of carboxylic acid used during the synthesis. Based on the shape of the isotherms, the microporous/supermicroporous and mesoporous natures of the materials can be clearly differentiated. The isotherms for MS-CA and MS-TA can be categorized as Type IV with an H2-type hysteresis loop (Figure 3), characteristic of ink-bottle-like mesopores. Both exhibit wide pore size distributions. In contrast, the silica xerogels prepared with ascorbic acid show Type I isotherms, indicating microporous characteristics. The MS-MdA sample displays a transitional behavior between micro- and mesoporous structures, with a Type I(b) isotherm and the formation of supermicropores (0.7–2 nm). The MS-MA and MS-OA samples also exhibit mixed characteristics, showing Type I(b) isotherms associated with supermicropores, along with a small H2-type hysteresis loop, indicative of ink-bottle-like pores with narrow pore entrances [31].

The effects of the different types of organic acids were more apparent when compared with samples prepared without any acid or with a strong mineral acid, such as nitric acid. In the former case (MS-TEOS), a well-developed mesoporous structure with a narrow pore size distribution was formed, characterized by an H2-type hysteresis loop and an average pore size of approximately 8 nm. In contrast, the use of nitric acid (MS-HNO_3_) results in a xerogel containing predominantly supermicropores. This indicates that a higher acidity of the synthesis mixture leads to faster hydrolysis but slower condensation of the silica precursor, which favors linear/chain-like polymer structures rather than highly cross-linked three-dimensional clusters. Because the polymer chains are less branched and more flexible, the gel skeleton shrinks more during aging and drying, leading to the formation of smaller pores [2,7,32]. In the acid-catalyzed formation of silica xerogels, rapid hydrolysis and slow condensation steps lead to the development of an open network structure, which undergoes more intensive shrinkage than the highly cross-linked structures typically formed during base-catalyzed synthesis. From this perspective, the intrinsic acidity of carboxylic acids plays a decisive role, resulting in pore structures similar to those obtained using nitric acid. Drying control or templating effects are either absent or only minimally detectable in most cases. As nitric acid is a much stronger acid than the carboxylic acids used in this study, its application is expected to result in higher microporosity in the corresponding silica xerogel. However, the high water-to-TEOS ratio also leads to a diluted alkoxide concentration and a slower gelation rate. From an environmental standpoint, the use of carboxylic acids is advantageous because of their benign recovery and low ecological impact. Notably, a porogenic effect was observed only in the samples prepared with citric or tartaric acid.

When comparing key textural properties, such as the total and micropore volumes (Table 3), it is evident that the samples prepared with tartaric and citric acid do not contain micropores but exhibit the highest total pore volumes among all the preparations.

Citric acid has been found to function as a template, as demonstrated by Takahashi et al. [33]. This behavior is attributed to its high solubility in water and the weak interaction of its carboxylic functional groups with surface silanol (Si–OH) groups via hydrogen bonding. Citric acid can mix homogeneously with hydrated silica oligomers at the nanometer scale to form an organic–inorganic composite. It occupies space within the developing silica network and inhibits shrinkage during drying. However, because of its weak interaction with silica, it does not hinder the further condensation of the silanol groups. As a result, the silica network becomes sufficiently rigid to form a highly porous gel structure. A similar phenomenon may occur in the case of tartaric acid, although its porogenic influence becomes evident only at higher concentrations or elevated drying temperatures (see Figure 1 and Figure 2) during the formation of a mesoporous system. This may be related to its different behaviors in aqueous solutions, specifically its dissociation into tartrate anions, the extent of solvation, and hydrogen bonding with water molecules. Although the pKa_1_ values of citric and tartaric acids are similar (CA: 3.13; TA: 2.98), tartaric acid contains only two carboxylic acid groups and has a much lower water solubility than citric acid (59 vs. 13.9 g/100 mL water), which may limit its templating efficiency under standard synthesis conditions.

In our synthesis method, no prehydrolysis of TEOS was performed; therefore, we expect that the intrinsic acidity of the acids influences the formation of the xerogel. The stronger the acidity, the higher the extent of hydrolysis and the formation of silica mono- and oligomers. From that point of view, all the synthesis mixtures showed pH values between 1.1 and 1.75 except the ascorbic acid with 2.4. These results can explain the correlations between the pH of the solution and the mesopore surface area of the xerogels formed. The MS-AA-60-1-C sample exhibited a purely microporous structure. The pH of the synthesis mixture was near the isoelectric point of silica, resulting in the formation of a suspension with a neutral or slightly negatively charged surface, under which conditions the formation of a mesoporous framework is unlikely [34,35]. The hydroxyl groups of ascorbic acid are less acidic than those of other carboxylic acids, leading to minimal interaction with the silica matrix. Consequently, ascorbic acid does not act as an effective porogen; however, it may exert some drying control effect responsible for the slightly higher specific surface area compared to the control sample synthesized in the absence of any acid (MS-TEOS-60-1-C).

#### 3.1.4. Thermogravimetric Analysis and FTIR Spectra

**Figure 4:**
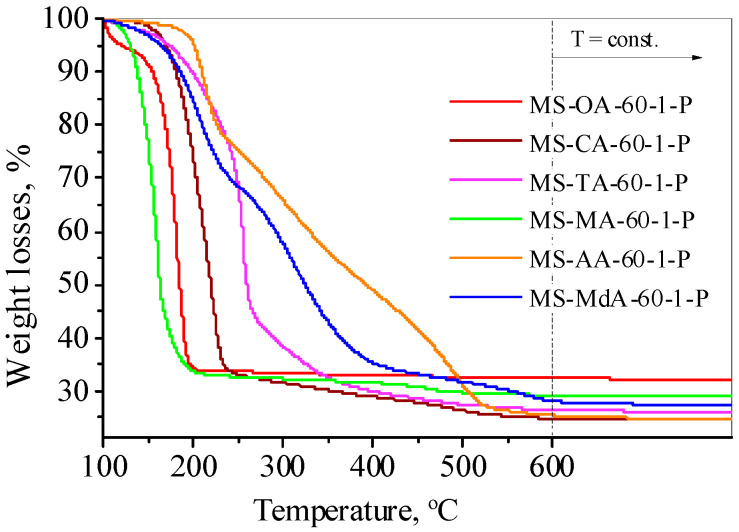
TPO of as-synthesized organic acid-containing silica xerogels (acid: TEOS = 1, dried at 60 °C).

Information on the interactions between silica and the acids in the composites can be obtained from the thermogravimetric curves of the organic-containing xerogels. The complete removal of organic acids occurred at different temperatures (Figure 4). From the weight loss–temperature profiles, it can be observed that, in most cases, the decomposition temperature of the organic acid in the silica–acid composite was higher than that of the pure acid (175–200 °C–CA; 170–220 °C–TA; 180–200 °C–MdA; 130 °C–MA; 157 °C-OA). It is characteristic of all carboxylic acids that their thermal decomposition starts before the total melting; thus, no precise melting point can be determined for them. We suggest that the higher decomposition temperatures detected in silica-organic composites with AA and MdA indicate stronger interactions between TEOS and these acids. Citric and tartaric acid-containing silica materials represent a transition between very weak and very strong interaction forces.

**Figure 5:**
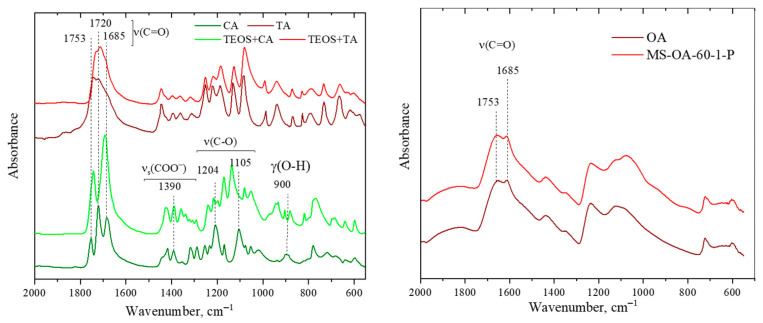
ATR-FRIT spectra of MS-CA-60-1-P, MS-TA-60-1-P, and MS-OA-60-1-P and the corresponding pure car.

To investigate the interaction between TEOS and some of the acids used, ATR-FTIR spectroscopy was performed. The obtained spectra are shown in Figure 5.

The FT-IR spectra of citric and tartaric acids displayed strong bands in the ~1750–1680 cm^−1^ region, which were assigned to the C=O stretching vibrations of protonated carboxylic acid groups. Strong intermolecular hydrogen bonding in the solid state broadens and shifts the C=O stretch, as observed in the interaction with silica nanoparticles. The band at ~1390 cm^−1^ corresponds to the symmetric stretching of carboxylate groups, indicating partial ionization and hydrogen bonding. The region 1250–1050 cm^−1^ is dominated by the C–O stretching vibrations. The changes upon interaction with silica indicate that citric and tartaric acids are highly distributed in the silica matrix and are bonded through the carbonyl groups rather than the hydroxyl groups of the respective acid.

In contrast, the interaction of oxalic acid with silica is less pronounced, and only the ratio of carbonyl bands changes, indicating that oxalic acid is connected to the silanol groups via weak hydrogen bonds. The similarity between the spectra of pure crystalline oxalic acid and the silica-modified variety indicates that most of the oxalic acid remained in the crystalline state in an H-bonded network after interaction with silica.

Overall, the FT-IR results demonstrate that the nature and strength of the interaction between organic acids and silica strongly depend on the molecular structure of the acid. Citric and tartaric acids exhibit stronger carbonyl-mediated interactions and more homogeneous dispersion within the silica matrix, whereas oxalic acid shows weaker interactions and largely preserves its crystalline character.

#### 3.1.5. Solid State NMR Investigation of Silica Xerogels

**Figure 6:**
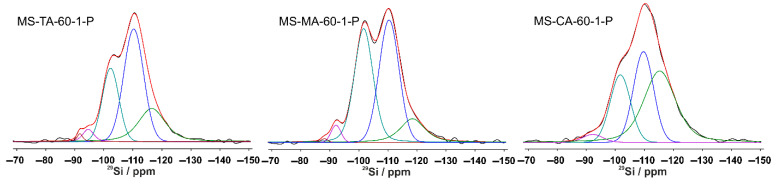
Experimental (black) and simulated (red) direct-excitation ^29^Si NMR spectra of the studied materials before template removal. The individual contributions of the different Si environments are represented by colored lines.

Solid-state NMR spectroscopy was used to characterize the samples before and after acid removal to study the effect of the acid type on the structural characteristics of mesoporous silica. The chemical shifts and relative intensities of the resonances in the direct-excitation ^29^Si NMR spectra provide valuable information about the nature and quantitative distribution of the different (SiO)_n_Si(OH)_4−n_ species (n = 1, 2, 3; i.e., Q^n^ species) in the silica matrix. The ^29^Si NMR spectra of all the MS materials exhibited a similar pattern, consisting of four partially overlapping resonances. As a representative case, Figure 6 shows the direct-excitation ^29^Si spectra of the acid-containing MS-MA-60-1-P, MS-TA-60-1-P, and MS-CA-60-1-P samples, while for the sake of clarity, the direct-excitation ^29^Si spectra for the rest of the samples, along with all ^1^H⟶^29^Si CPMAS spectra, are presented in Figures S2–S4. In all cases, a pair of resonances appears in the range of –110 to –120 ppm, which is typical for Q^4^ species [Si(0OH) units] that represent the main building blocks of the silicate framework [36,37,38,39]. The principal resonance for the Q^4^ species in MS materials is usually centered near −111 ppm. The additional high-field resonance in this region reflects the structural heterogeneity within the Q^4^ environment, most likely associated with distorted bond lengths and bond angles induced by the incorporation of organic acid molecules. The resonance observed at approximately −101 ppm is characteristic of Q^3^ species [Si(1OH) units], while the weaker signal at around −92 ppm corresponds to a minor fraction of Q^2^ structures [Si(2OH) units], which was also confirmed by ^1^H⟶^29^Si CPMAS experiments (Supporting Information, Figures S3 and S4). The Q^3^ and Q^2^ species are generally attributed to defect sites located on the silica surface and/or pore edges. Quantitative analysis of the spectral components, performed through deconvolution of the ^29^Si resonances, allowed estimation of the distribution of Q^n^ species, as summarized in Table 4. The results revealed that, regardless of the organic acid used, the relative fractions of the different Q^n^ species remained within a comparable range, with only minor variations.

Across all samples, the total Q^4^ fraction was consistently between 59% and 70%, whereas the combined Q^3^ + Q^2^ fraction ranged from 43% to 26%. In some cases (MS-TA-60-1-P and MS-MA-60-P), the formation of a trace amount of Q^1^ species [Si(3OH)] was also detected.

**Figure 7:**
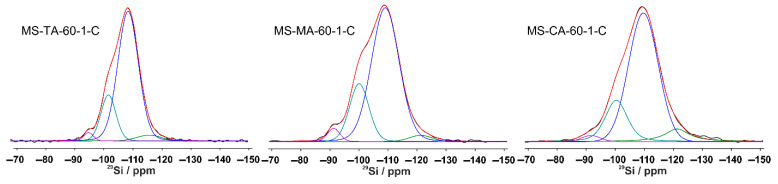
Experimental (black) and simulated (red) direct-excitation ^29^Si NMR spectra of the selected materials after template removal. The individual contributions of the different Si environments are represented by colored lines.

Analysis of the direct-excitation ^29^Si spectra of the samples subjected to template removal by calcination at 550 °C indicates that this treatment induced structural modifications within the silica framework. In all the calcined materials, the proportion of Q^4^ units increased to 72–79%, accompanied by a corresponding decrease in the fraction of silanol-containing species (Q^3^ + Q^2^), which decreased to 28–21% (Table 4 and Figure 7 and Figure S2). Notably, the type of organic acid employed as a template had no significant influence on the structural characteristics of the materials after calcination.

The predominance of Q^4^ structures highlights the high degree of crosslinking in the silica network. The reduction in Q^3^ and Q^2^ species, coupled with the increase in Q^4^ units, strongly suggests that calcination promoted further consolidation of the framework. This transformation is attributed to the thermal condensation of silanol groups, resulting in the formation of a highly interconnected silicate network with enhanced structural rigidity.

#### 3.1.6. Morphology of Silica Xerogels Prepared by Carboxylic Acids

**Figure 8:**
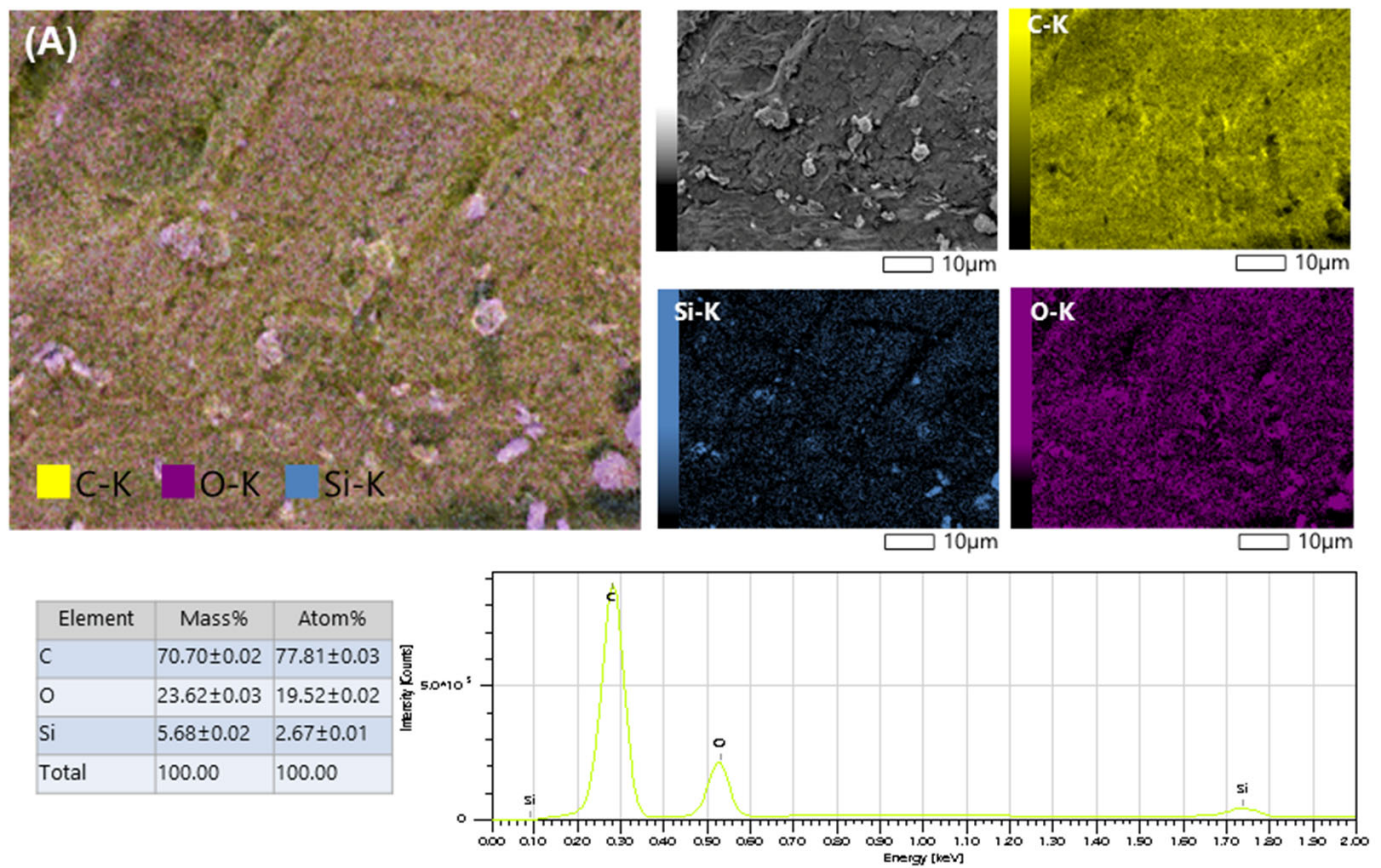

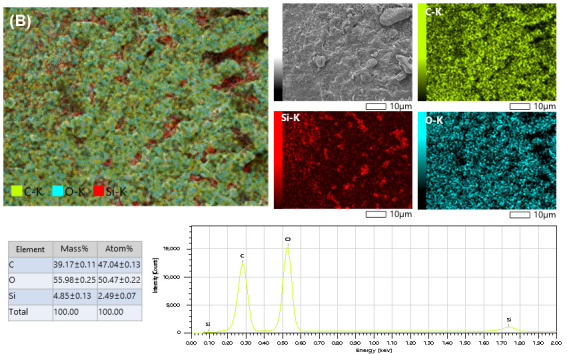
SEM-EDS mapping of the element distribution on the surface layer of the organic template–containing samples: (**A**) citric acid and (**B**) tartaric acid.

Scanning electron microscopy coupled with energy-dispersive X-ray spectroscopy (SEM–EDS) mapping was employed to examine the elemental distribution within the surface layer of the organic template–containing samples. To compare the significance of the acid type on the textural properties of the obtained silica, citric acid- and tartaric acid-containing samples, illustrated in Figure 8A,B, respectively, were investigated. For both samples, the organic components were relatively homogeneously dispersed throughout the silica matrix; however, a higher dispersion of carbonaceous material was observed for citric acid. The absence of sufficiently large carboxylic acid crystallites indicates that the entrapment of organic molecules within the silica gel matrix effectively inhibits their agglomeration. Instead, this encapsulation promotes the formation of smaller crystallites, which may act as porogenic agents.

Scanning electron microscopy (SEM) images (Supporting Information, Figure S5) of MS-CA-60-1-P and MS-TA-60-1-P reveal the formation of a porous structure and the specific organization of the acid-silica composites, which play an important role in the morphology of silica materials. The SEM images (Supporting Information, Figure S6) of the calcined samples show the typical morphology of mesoporous silica materials with a non-ordered structure. Macroscopically, all materials are similar and are built of fragmented blocks. The lack of well-defined morphology and uniform particle shapes or sizes suggests that the organic acid templates influenced the development of the porous structure within the silica matrix but did not play a significant role in directing the morphology or shaping of the particles.

**Figure 9:**
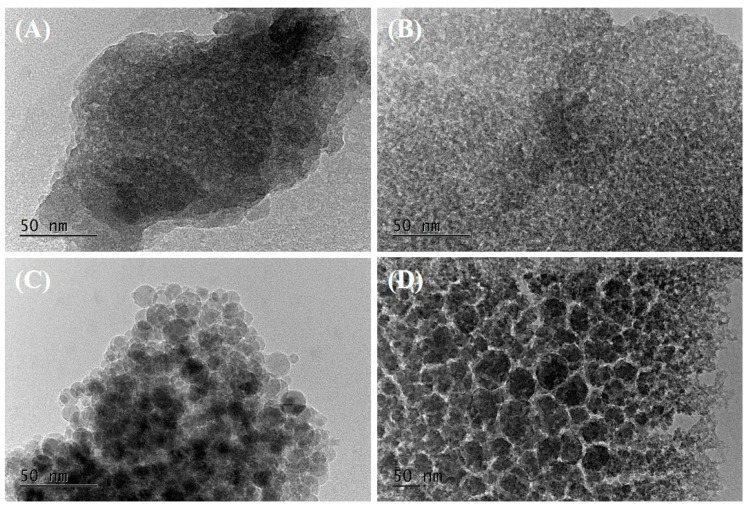
Bright-field transmission electron microscopy micrographs of calcined silica xerogels prepared with different carboxylic acids: (**A**) MS-OA-60-1-C, (**B**) MS-MA-60-1-C, (**C**) MS-CA-60-1-C, (**D**) MS-TA-60-1-C.

TEM analysis of silica xerogels can provide information about the morphology, microstructure, and pore system of the samples. The samples prepared with OA or MA showed typical images of amorphous silica materials with the formation of large features without any particular shape (Figure 9A,B). In addition, spherical particles with diameters of 10–15 nm for CA and areas with large pores with diameters of 40–50 nm for TA were clearly observed (Figure 9C,D). According to the BF TEM micrographs, silica has a non-ordered, wormhole-like pore system, but in the case of MS-CA-60-1-C, the interparticle voids are also part of the pore system, as supported by the nitrogen physisorption data.

#### 3.1.7. Optimization of the Template Removal Procedure

In pursuit of a sustainable, environmentally friendly, and energy-efficient approach for producing mesoporous silicates, the organic content of the composites was eliminated using two techniques: high-temperature calcination and water extraction. The conventional method for organic matter removal, calcination, was performed at 550 °C for 5 h. In contrast, the extraction of the organic phase was carried out under significantly milder conditions, specifically 1 h of stirring at room temperature in aqueous media. Full extraction was achieved in two cycles, and the resulting samples were designated as “W”. Organic removal was investigated in samples prepared using an acid-to-TEOS molar ratio of 1 and dried at 60 °C. Unlike calcination, the aqueous extraction method enables the recovery and potential reuse of organic acids, which would otherwise be completely decomposed during high-temperature treatments. The successful removal of acids by extraction and calcination was confirmed by TG investigations in air treatment up to 600 °C, as presented in Figure S7. The results show around 5 wt. % of water removal at temperatures below 150 °C, whereas the weight losses above this temperature are negligible and are due to the dihydroxylation process in both samples, calcined and washed.

**Figure 10:**
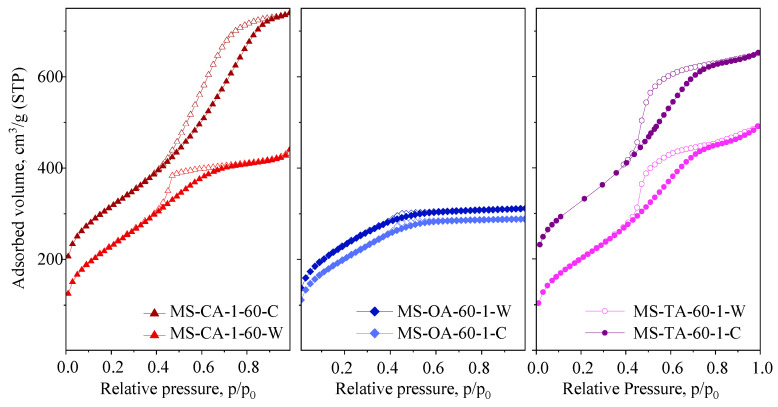
Nitrogen physisorption isotherms of silica xerogels prepared with an acid-to-TEOS molar ratio of 1, dried at 60 °C, and subjected to either calcination (C) or water extraction (W). The isotherms of the MS-CA-1-60-C and MS-TA-60-1-W samples were shifted by 100 cm^3^/g.

The materials prepared via the extraction method (Table 5 and Figure 10) exhibited superior textural characteristics compared to their calcined counterparts. For the MS-OA-60-1-W and MS-TA-60-1-W samples, the specific surface area increased by approximately 100 m^2^/g, and the pore volume increased by about 0.1 cm^3^/g. However, notable differences were observed in the isotherms of the MS-CA-60-1 sample (Figure 9) between the calcined and water-extracted versions. The water-extracted MS-CA-60-1-W sample exhibited a 40% lower pore volume, and the larger pores disappeared. This suggests that condensation of the silica matrix occurs not only during gelation and drying, but predominantly during the calcination step. Omitting calcination results in higher shrinkage of the silica networks.

MS-CA-60-1-P differs in that a significantly higher amount of citric acid can be incorporated into the silica matrix compared to other carboxylic acids. This finding was supported by the observations of Takahashi et al. [33], who reported that the high solubility of citric acid contributes to the swelling of the wet gel and the formation of an amorphous CA–silica nanocomposite. High-temperature calcination led to the shrinkage of the silica network and dehydroxylation of the surface silanol groups, as confirmed by NMR analysis (Table 4).

This resulted in the formation of a more condensed silica matrix with fewer surface groups and a lower specific surface area. In contrast, the other two xerogels exhibited the opposite behavior, as expected. According to the thermogravimetric results, the interaction of tartaric acid with the silica oligomers was slightly stronger but should be more specific and limited than that of citric acid. Thus, the condensation process can proceed to a greater extent during the sol-gel process, and no shrinkage occurs due to water extraction. These findings demonstrate that the energy-intensive calcination process can be effectively replaced by water, a non-toxic and environmentally benign solvent. The carbon content of the water-extracted samples was assessed using temperature-programmed oxidation (TPO) analysis, which indicated that the observed weight loss corresponded solely to the dehydroxylation of silica above 100 °C. The simple washing procedure successfully removed all organic acids, allowing their regeneration and reuse.

#### 3.1.8. Regeneration of the Organic Acids

**Figure 11:**
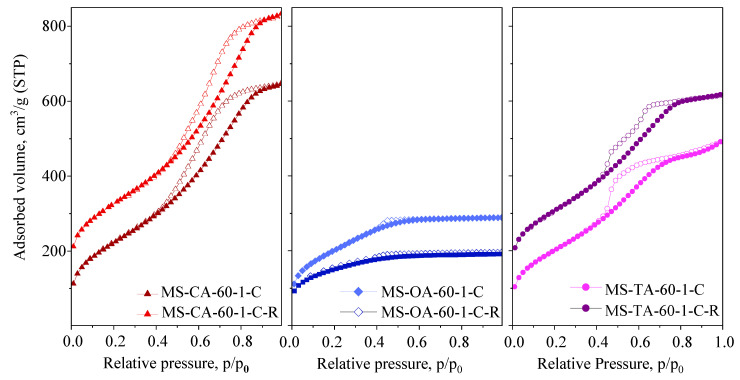
Nitrogen physisorption isotherms of silica xerogels (TEOS: acid = 1, dried at 60 °C): comparison between calcined samples (C) and samples prepared using recovered acids under identical synthesis conditions (C-R). The isotherms of the MS-CA-60-1-C-R and MS-TA-60-1-C-R samples were shifted by 100 cm^3^/g.

Following the extraction procedure, the acid was regenerated by collecting the filtrate obtained after silica precipitate separation and subsequently drying it at 60 °C. The results demonstrate that more than 95% of the initially applied acid can be recovered through filtration and drying. XRD patterns of the recrystallized acids were identical to those of the parent compounds, indicating their suitability for reuse in subsequent synthesis cycles (Supplementary information, Figure S1B). The silica materials obtained using the recycled acids exhibited similar or even improved physicochemical properties compared to those prepared using fresh acids. Figure 11 shows the nitrogen physisorption isotherms of the samples synthesized using recycled citric, oxalic, and tartaric acids. Corresponding textural parameters are summarized in Table 6.

The results show that the obtained mesoporous silicas could be prepared by a simple and economically efficient procedure, making them suitable for different applications, such as adsorption, drug delivery, and catalysis.

## 4. Conclusions

A novel method was developed for the synthesis of porous silica xerogels using different carboxylic acids as non-surfactant, eco-friendly porogens. The influence of the synthesis parameters and the nature of the applied acids on the textural and structural properties of the final materials was investigated. Citric and tartaric acids had a favorable effect on the formation of mesoporous silica, whereas the other acids led to the formation of microporous silica. An optimal medium strength of silica interaction with carboxylic acids is required to build well-ordered mesoporous silica. Tartaric acid showed a similar favorable effect in the regulation of the pore system as citric acid; however, only at higher acid concentrations owing to its lower solubility and differences in its chemical nature. Well-ordered mesoporous silica with a uniform pore size was prepared using tartaric acid, and the pore size was more precisely controlled by the drying temperature. Template removal via water extraction yielded silica materials with lower specific surface areas and pore volumes for citric acid than conventional high-temperature calcination. However, tartaric acid had a beneficial effect because of its slightly stronger but more specific and limited interaction with silica oligomers. Environmentally friendly template removal via water extraction enabled the recovery and reuse of organic acids with over 95% efficiency. Our results confirm that tartaric acid can be efficiently and economically applied for the controlled synthesis of mesoporous silica suitable for different applications such as adsorption, drug delivery, or catalysis, which will be the focus of our further investigations.

## Figures and Tables

**Table 1:** Textural properties of silica xerogels prepared using citric, oxalic, and tartaric acids (Acid: TEOS = 3) dried at different temperatures.

Samples	SSA m^2^/g	V_tot_ cm^3^/g	SA_mes_ m^2^/g	V_meso_ cm^3^/g	PD nm
MS-CA-30-3-C	763	1.08	763	1.08	-
MS-CA-60-3-C	681	1.01	681	1.01	3.9/6.7
MS-CA-90-3-C	601	1.17	601	1.17	4.6/8.6
MS-OA-30-3-C	696	0.50	696	0.50	-
MS-OA-60-3-C	705	0.53	687	0.53	-
MS-OA-90-3-C	753	0.67	753	0.67	-
MS-TA-30-3-C	782	0.82	782	0.82	-
MS-TA-60-3-C	692	0.96	692	0.96	4.6
MS-TA-90-3-C	667	1.12	667	1.12	5.6

SSA—specific surface area calculated using the BET equation; V_tot_—total pore volume; SA_mes_—mesopore surface area, calculated using the t-plot method; V_meso_—mesopore volume; PD—maximum pore diameter calculated using the BJH method, and in the case of materials with a bimodal pore size distribution, two values are separated by a slash.

**Table 2:** Textural properties of silica xerogels prepared with different acid-to-TEOS molar ratios and dried at 60 °C.

Samples	SSA m^2^/g	V_tot_ cm^3^/g	SA_mes_ m^2^/g	V_meso_ cm^3^/g	PD nm
MS-CA-60-1-C	814	1.00	814	1.00	3.9/5.4
MS-CA-60-3-C	681	1.23	681	1.23	3.9/6.7
MS-OA-60-1-C	730	0.45	730	0.45	2.5
MS-OA-60-3-C	705	0.53	705	0.53	-
MS-TA-60-1-C	738	0.76	738	0.76	-
MS-TA-60-3-C	692	0.96	692	0.96	4.6

SSA—specific surface area calculated using the BET equation; V_tot_—total pore volume; SA_mes_—mesopore surface area, calculated using the t-plot method; V_meso_—mesopore volume; PD—maximum pore diameter calculated using the BJH method, and in the case of materials with a bimodal pore size distribution, two values are separated by a slash.

**Table 3:** Textural properties of silica xerogels prepared using different carboxylic acids (acid: TEOS = 1, drying at 60 °C).

Samples	SSA m^2^/g	V_tot_ cm^3^/g	SA_mes_ m^2^/g	V_mic_ cm^3^/g	V_meso_ cm^3^/g	PD nm
MS-CA-60-1-C	814	1.00	814	-	1.00	3.9/5.4
MS-OA-60-1-C	727	0.45	727	-	0.45	2.5
MS-AA-60-1-C	504	0.24	-	0.24	0.00	-
MS-MA-60-1-C	923	0.52	857	0.02	0.50	3.1
MS-TA-60-1-C	738	0.76	738	-	0.76	-
MS-MdA-60-1-C	802	0.43	627	0.08	0.35	3.2
MS-TEOS-60	422	0.69	422	-	0.00	7.9
MS-HNO_3_-60	781	0.44	745	0.01	0.43	3.1

SSA—specific surface area calculated by the BET equation; V_tot_—total pore volume; SA_mes_—mesopore surface area, calculated by the t-plot method; V_mic_—micropore volume calculated by the t-plot method; V_meso_—mesopore volume; PD—maximum pore diameter calculated by the BJH method, and in the case of materials with a bimodal pore size distribution, two values are separated by a slash.

**Table 4:** ^29^Si NMR data of organic acid-containing (P) and calcined (C)silica xerogels (acid: TEOS = 1, drying at 60 °C).

Samples	Si(3OH) %	Si(2OH) %	Si(1OH) %	Si(0OH) %	Si(0OH) %
MS-AA-60-1-P	-	10	26	40	24
MS-AA-60-1-C	-	2	21	76	1
MS-TA-60-1-P	1	4	25	47	23
MS-TA-60-1-C		2	19	75	4
MS-MA-60-1-P	1	3	40	43	13
MS-MA-60-1-C	-	3	22	72	3
MS-MdA-60-1-P	-	2	39	42	17
MS-MdA-60-1-C	-	3	20	75	2
MS-CA-60-1-P	-	3	23	30	44
MS-CA-60-1-C	-	2	20	70	8
MS-OA-60-1-P	-	3	29	51	17
MS-OA-60-1-C	-	2	26	67	5

**Table 5:** Textural properties of silica xerogels synthesized using citric, oxalic, and tartaric acids (TEOS: acid = 1, dried at 60 °C). Organic matter removal was performed using either calcination (C) or water extraction (W).

Samples	SSA m^2^/g	V_tot_ cm^3^/g	SA_mes_ m^2^/g	V_mic_ cm^3^/g	V_meso_ cm^3^/g
MS-CA-60-1-C	814	1.00	814	-	1.00
MS-CA-60-1-W	829	0.62	829	-	0.62
MS-OA-60-1-C	727	0.45	706	0.02	0.43
MS-OA-60-1-W	855	0.56	822	0.02	0.54
MS-TA-60-1-C	738	0.76	738	-	0.76
MS-TA-60-1-W	831	0.86	831	-	0.86

SSA—specific surface area calculated using the BET equation; V_tot_—total pore volume; SA_mes_—mesopore surface area, calculated using the t-plot method; V_mic_—micropore volume calculated using the t-plot method; V_meso_—mesopore volume.

**Table 6:** Textural properties of silica xerogels (acid: TEOS = 1, dried at 60 °C, calcined (C)) prepared using original or recovered (C-R) acids.

Samples	SSA m^2^/g	V_tot_ cm^3^/g	SA_mes_ m^2^/g	V_mic_ cm^3^/g	V_meso_ cm^3^/g	PD nm
MS-CA-60-1-C	814	1.00	814	-	1.00	3.9/5.4
MS-CA-60-1-C-R	846	1.15	846	-	1.15	4.2/5.4
MS-OA-60-1-C	727	0.45	687	0.02	0.43	-
MS-OA-60-1-C-R	558	0.3	317	0.1	0.20	-
MS-TA-60-1-C	738	0.76	738	-	0.76	-
MS-TA-60-1-C-R	784	0.81	784	-	0.81	3.5/5.3

SSA—specific surface area calculated by the BET equation; V_tot_—total pore volume; SA_mes_—mesopore surface area, calculated by the t-plot method; V_mic_—micropore volume calculated by the t-plot method; V_meso_—mesopore volume; PD—maximum pore diameter calculated by the BJH method, and in the case of materials with a bimodal pore size distribution, two values are separated by a slash.

## Data Availability

The original contributions presented in this study are included in the article/Supplementary Materials. Further inquiries can be directed to the corresponding author.

## Supplementary Materials

The following supporting information can be downloaded at: https://www.mdpi.com/article/10.3390/nano16010045/s1, Table S1. ICDD card numbers used for identification of diffrerent acids. Table S2. Textural properties of silica xerogels prepared with citric acid using different acid: TEOS ratios and drying at 60 °C. Figure S1. High-angle XRD patterns of silica-organic acid composites (A) and XRD patterns of the recycled acids (B). Figure S2. Experimental (black) and simulated (red) direct excitation 29Si NMR spectra of the studied materials before (left) and after template removal (right). The individual contributions of the different Si environments are given with colored lines. Figure S3. 1H→29Si CPMAS (red) and direct excitation 29Si spectra (black) of the studied samples with templates. Figure S4. 1H→29Si CPMAS (red) and direct excitation 29Si spectra (black) of the studied samples after template removal. Figure S5. SEM images of MS-CA-60-1-P and MS-TA-60-1-P. Figure S6. SEM images of MS-CA-60-1-C, MS-OA-60-1-C and MS-TA-60-1-C. Figure S7. TG analysis of MS-CA-60-1-W, MS-CA-60-1-C.
