# Supplementary material for: Green Surfactant-Free Synthesis of Mesoporous Silica Materials via a Biomass-Derived Carboxylic Acid-Assisted Approach
Source: Nanomaterials (Basel). 2025 Dec 29;16(1):45. doi: 10.3390/nano16010045 (PMC12787774; doi:10.3390/nano16010045)
Supplement: Supplementary file 1 [file nanomaterials-16-00045-s001.zip › nanomaterials-4028290-supplementary.pdf]

Supplementary information

Green Surfactant-Free Synthesis of Mesoporous Silica Materials via a Biomass-Derived Carboxylic Acid-Assisted Approach

Ivalina Trendafilova[1], Stela Grozdanova[1,2], Ágnes Szegedi[3], Pavletta Shestakova[1], Yavor Mitrev[1,2], Bogdan Ranguelov[4], Daniela Karashanova[5], Margarita Popova[1,2]

[1] Institute of Organic Chemistry with Centre of Phytochemistry, Bulgarian Academy of Sciences, Sofia, 1113, Bulgaria

[2] National Centre of Excellence Mechatronics and Clean Technologies, 8 bul. Kliment Ohridski, Sofia, Bulgaria

[3] Institute of Materials and Environmental Chemistry, HUN-REN Research Centre for Natural Sciences, Magyar tudósok krt. 2, Budapest, 1117, Hungary

[4] Institute of Physical Chemistry, Bulgarian Academy of Sciences, 1113 Sofia, Bulgaria

[5] Institute of Optical Materials and Technologies, Bulgarian Academy of Sciences, 1113 Sofia, Bulgaria

Table S1. ICDD card numbers used for identification of diffrerent acids

Samples	Crystalline phases	ICDD card No.
**MS-AA-30-1-P**	Ascorbic acid	22-1560
**MS-AA-60-1-P**	Ascorbic acid	22-1560
**MS-CA-30-1-P**	Citric acid, more silica	16-1157
**MS-CA-60-1-P**	Citric acid	16-1157
**MS-MA-30-1-P**	Maleic acid	22-1763
**MS-MA-60-1-P**	Maleic acid	22-1763
**MS-MdA-30-1-P**	D-Mandelic acid	32-1750
**MS-MdA-60-1-P**	D-Mandelic acid	32-1750
**MS-OA-30-1-P**	Oxalic acid dyhidrate (OAD)	14-0832
**MS-OA-60-1-P**	Oxalic acid+OAD	14-0832, 20-1816
**MS-TA-30-1-P**	Tartaric acid, D-, L+ mixture	20-1901 (D-), 33-1883 (L+)
**MS-TA-60-1-P**	Tartaric acid, D-, L+ mixture, more L+	20-1901 (D-), 33-1883 (L+)

Table S2. Textural properties of silica xerogels prepared with citric acid using different acid: TEOS ratios and drying at 60 ℃.

**Samples**	**SSA** m^2/g	V_{tot} cm^3/g
MS-CA-60-1-C	814	1.00
MS-CA-60-3-C	681	1.23
MS-CA-60-6-C	521	1.13

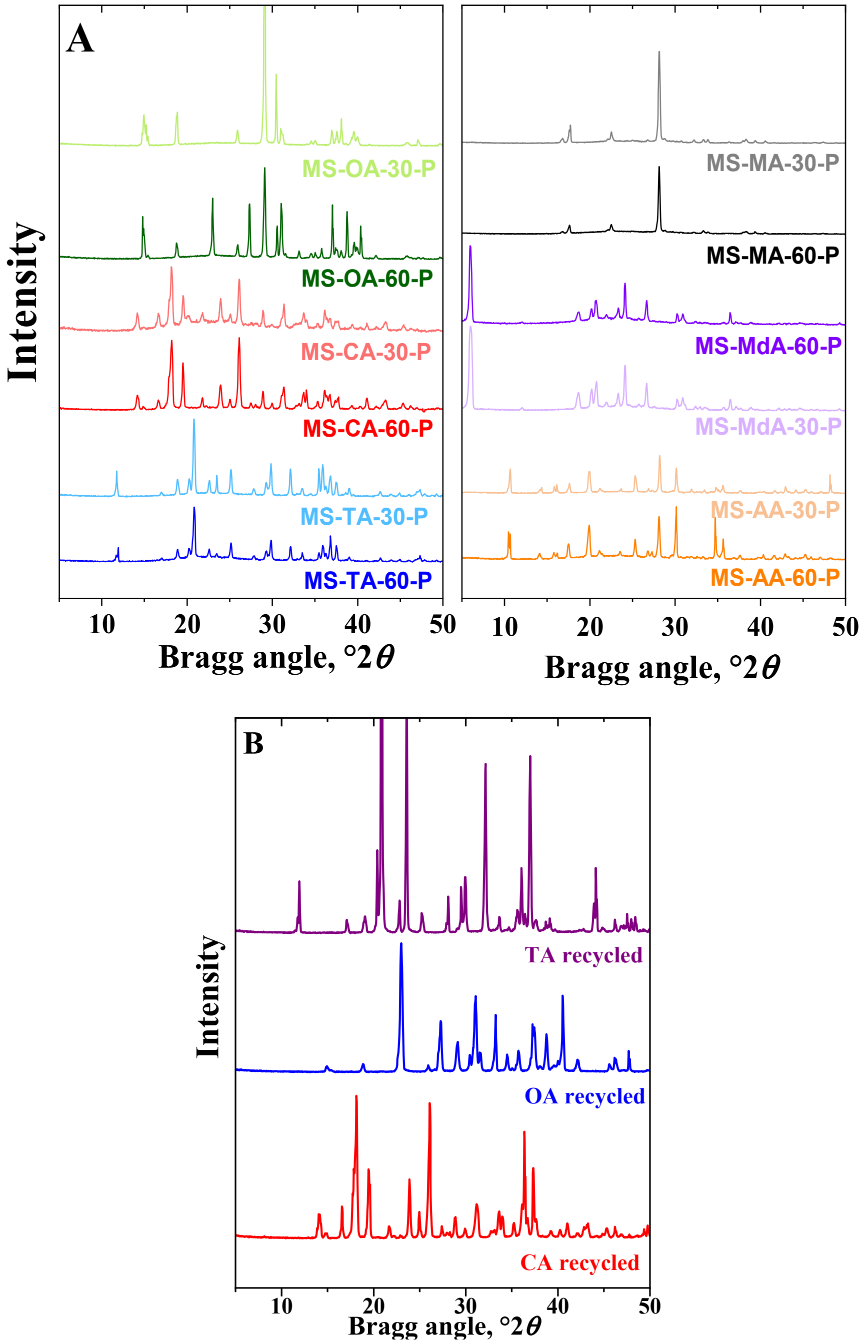

Figure S1. High-angle XRD patterns of silica-organic acid composites (A) and XRD patterns of the recycled acids (B).

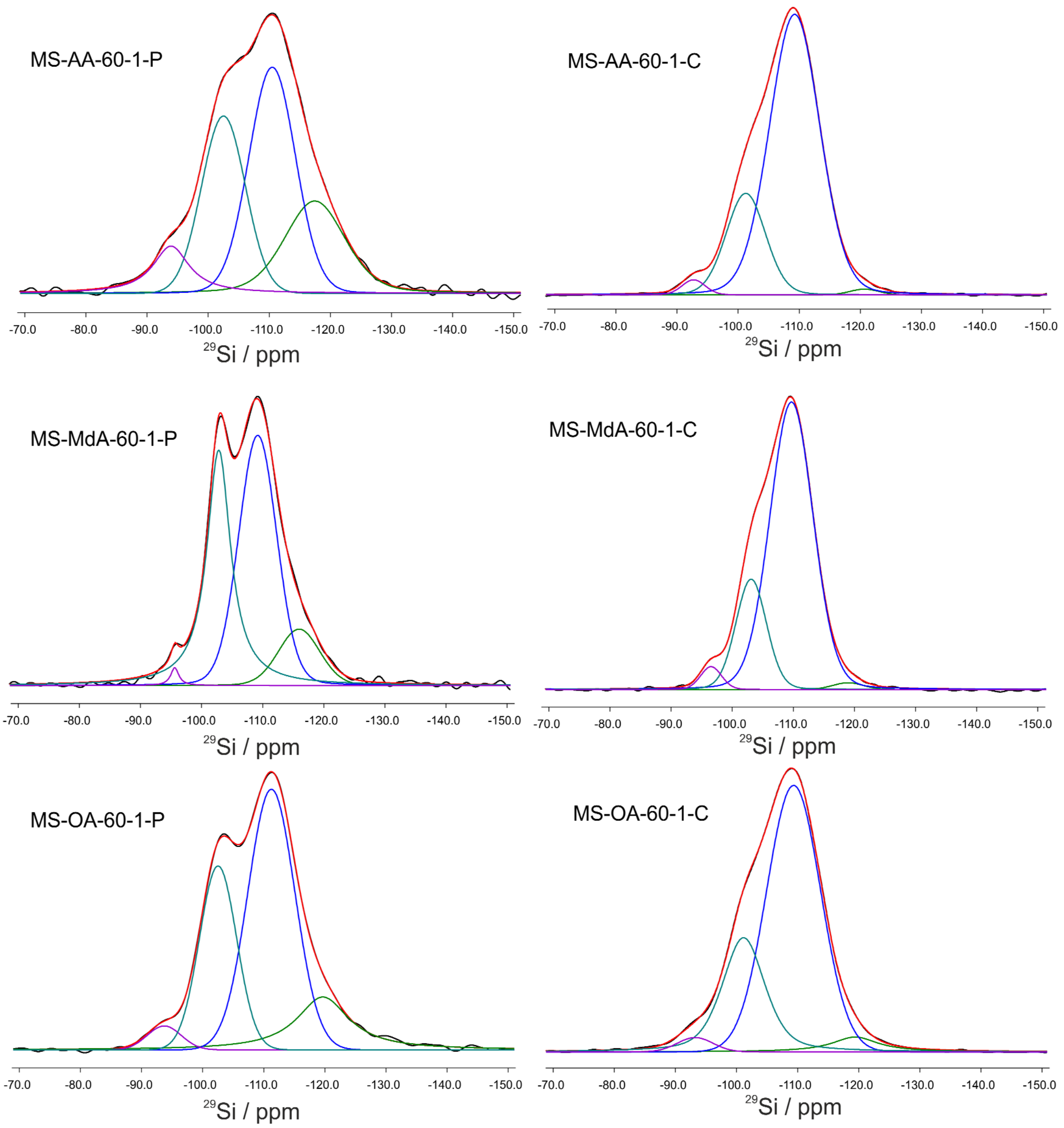

Figure S2. Experimental (black) and simulated (red) direct excitation ^{29}Si NMR spectra of the studied materials before (left) and after template removal (right). The individual contributions of the different Si environments are given with colored lines.

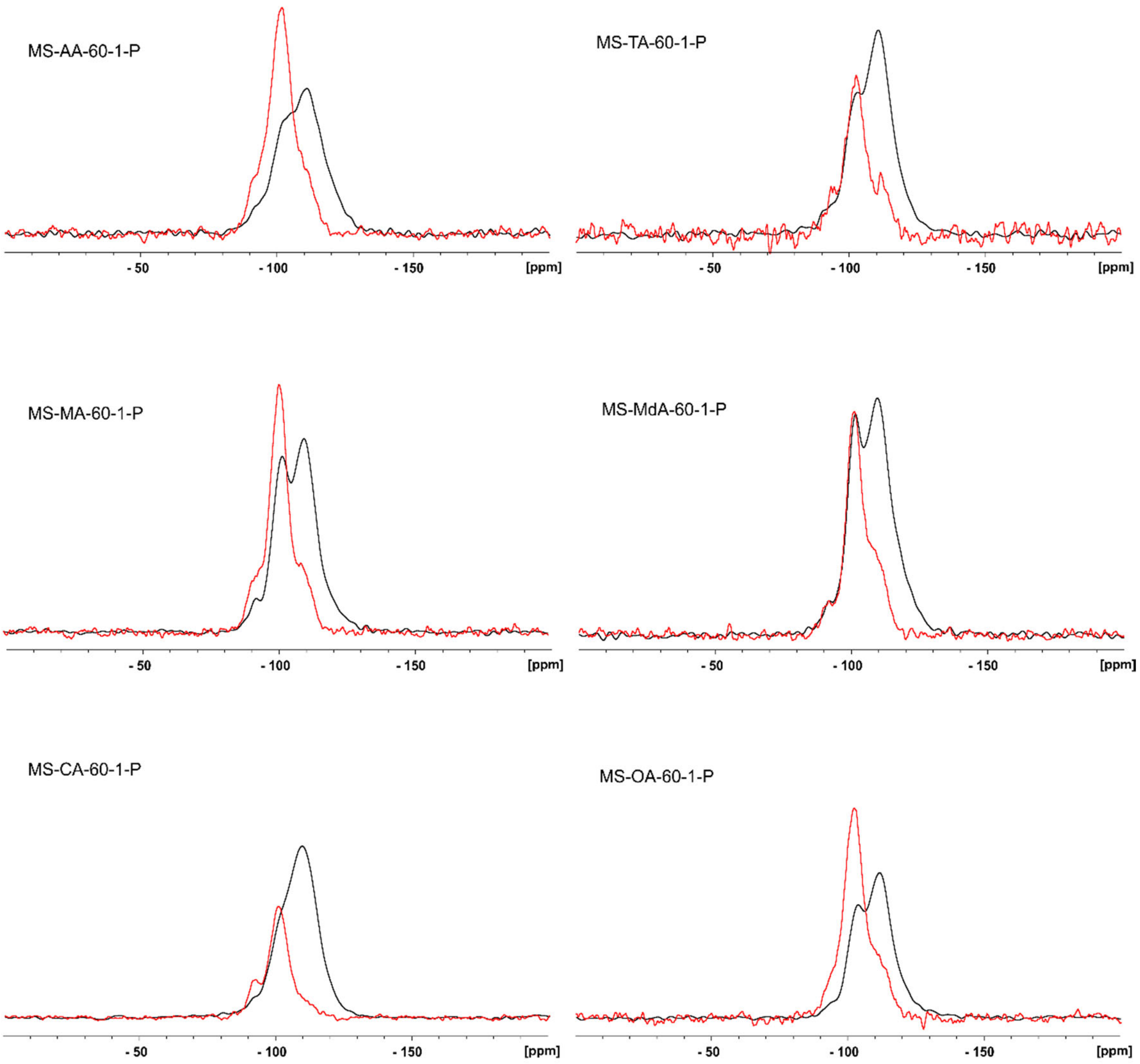

Figure S3. $^{1}H \rightarrow ^{29}Si$ CPMAS (red) and direct excitation ^{29}Si spectra (black) of the studied samples with templates.

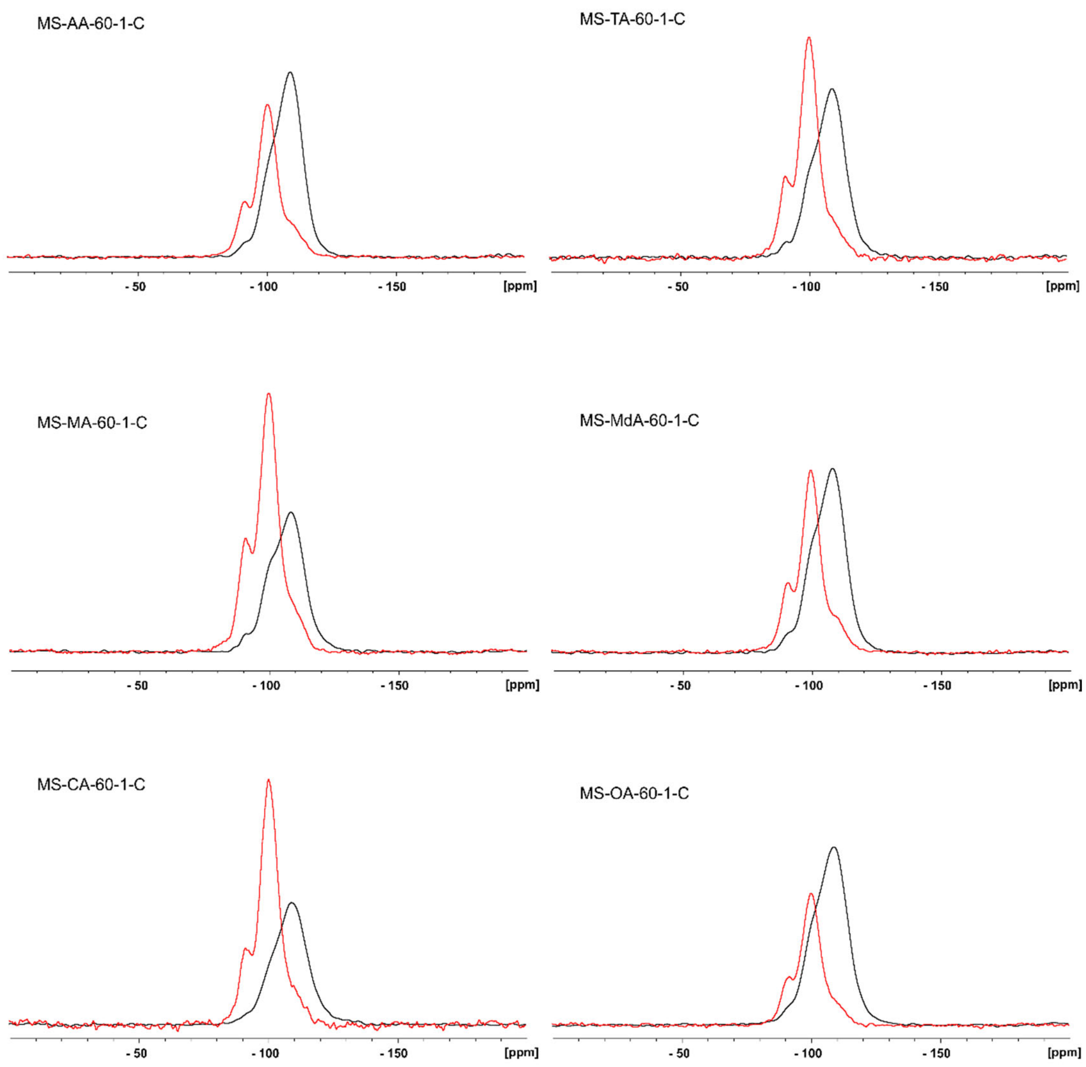

Figure S4. ${}^{1}H \rightarrow {}^{29}Si$ CPMAS (red) and direct excitation ${}^{29}Si$ spectra (black) of the studied samples after template removal.

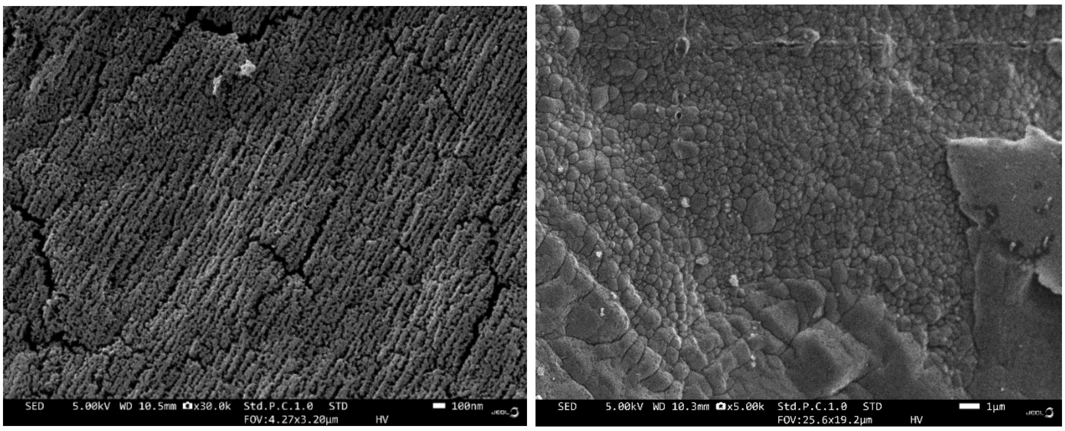

Figure S5. SEM images of MS-CA-60-1-P and MS-TA-60-1-P.

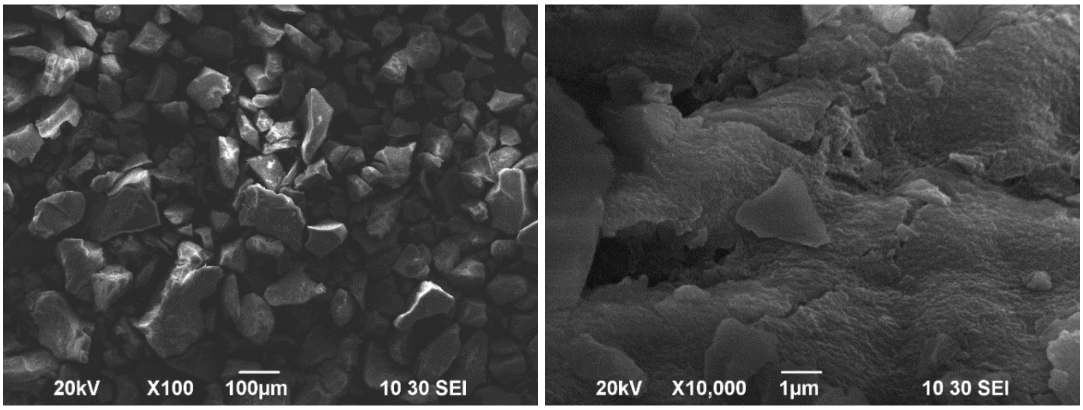

MS-CA-60-1-C

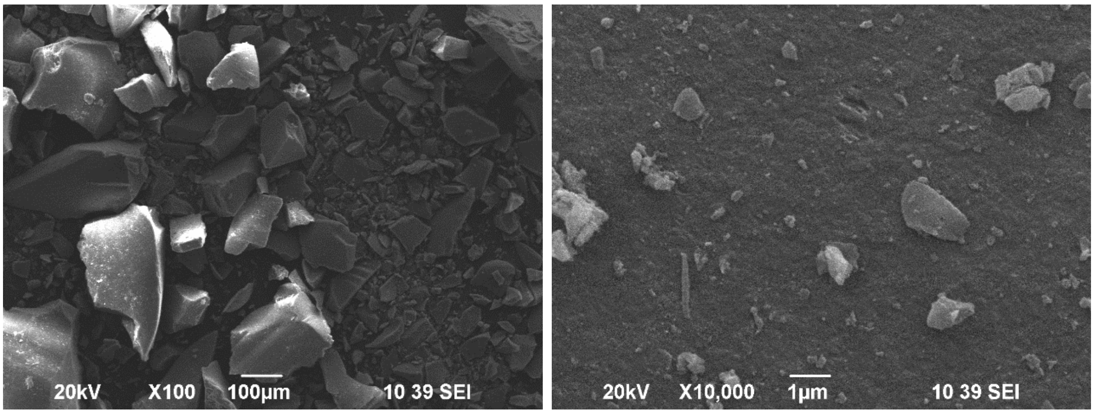

MS-OA-60-1-C

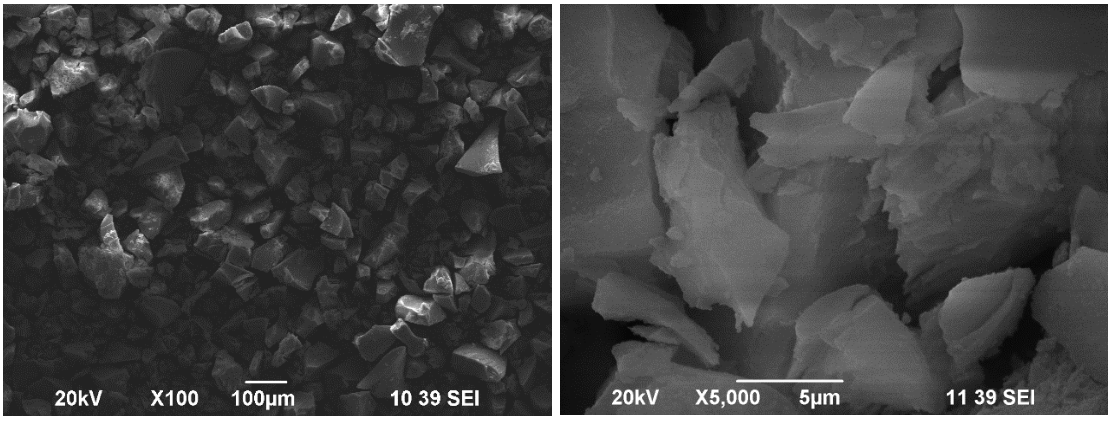

MS-TA-60-1-C

Figure S6. SEM images of MS-CA-60-1-C, MS-OA-60-1-C and MS-TA-60-1-C.

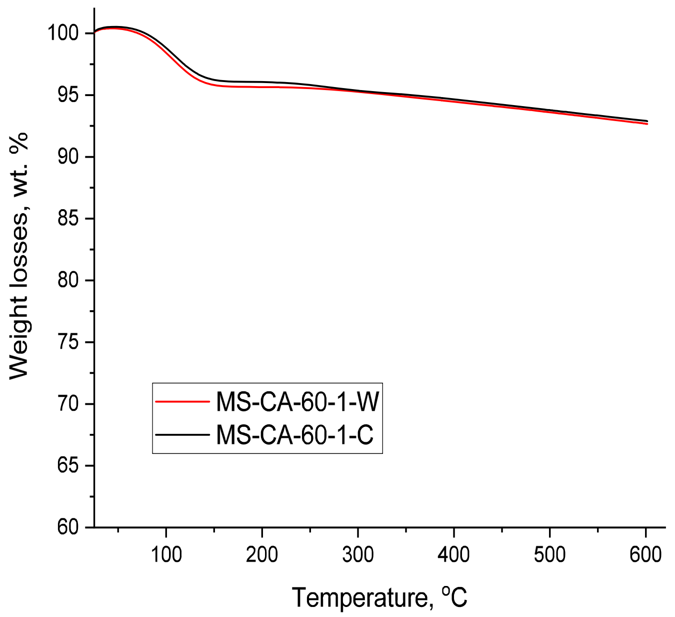

Figure S7. TG analysis of MS-CA-60-1-W, MS-CA-60-1-C
